# Macrophages—bone marrow mesenchymal stem cells crosstalk in bone healing

**DOI:** 10.3389/fcell.2023.1193765

**Published:** 2023-06-23

**Authors:** Siyu Fan, Xin Sun, Chuanchao Su, Yiwen Xue, Xiao Song, Runzhi Deng

**Affiliations:** ^1^ Department of Oral and Maxillofacial Surgery, Nanjing Stomatological Hospital, Affiliated Hospital of Medical School, Nanjing University, Nanjing, Jiangsu, China; ^2^ Central Laboratory of Stomatology, Nanjing Stomatological Hospital, Affiliated Hospital of Medical School, Nanjing University, Nanjing, Jiangsu, China

**Keywords:** bone healing, BMSCs, macrophages, inflammation, tissue regeneration

## Abstract

Bone healing is associated with many orthopedic conditions, including fractures and osteonecrosis, arthritis, metabolic bone disease, tumors and periprosthetic particle-associated osteolysis. How to effectively promote bone healing has become a keen topic for researchers. The role of macrophages and bone marrow mesenchymal stem cells (BMSCs) in bone healing has gradually come to light with the development of the concept of osteoimmunity. Their interaction regulates the balance between inflammation and regeneration, and when the inflammatory response is over-excited, attenuated, or disturbed, it results in the failure of bone healing. Therefore, an in-depth understanding of the function of macrophages and bone marrow mesenchymal stem cells in bone regeneration and the relationship between the two could provide new directions to promote bone healing. This paper reviews the role of macrophages and bone marrow mesenchymal stem cells in bone healing and the mechanism and significance of their interaction. Several new therapeutic ideas for regulating the inflammatory response in bone healing by targeting macrophages and bone marrow mesenchymal stem cells crosstalk are also discussed.

## 1 Introduction

Bone fracture is a common orthopaedic injury and a public health problem worldwide. According to statistics, there were approximately 178 million new fracture patients worldwide in 2019, an increase of 33.4% since 1990, resulting in a serious economic burden (Mills et al., 2017; Wu et al., 2021). Despite advances in fracture treatment, complications such as delayed bone union or nonunion still occur in 1.9%–4.9% of patients when there are large-scale bone defects, osteoporosis, fragility fractures in the elderly, and when patients have concomitant autoimmune or underlying disease, prolonging the recovery period while increasing the cost of patient care (Zura et al., 2016; Wildemann et al., 2021; Shapiro et al., 2022). Therefore, the promotion of bone healing has become a therapeutic priority in bone repair.

The intricate process of bone repair necessitates the collaboration of numerous systems. Inflammation plays a critical part in bone healing, which can be separated into three overlapping phases: inflammation, repair and remodelling (Loi et al., 2016; Moriarty et al., 2022). The inflammatory response in the ideal bone regeneration mode is finely regulated. However, incomplete bone healing happens when the acute inflammatory response is enhanced or suppressed, or when chronic inflammatory state persists (Claes et al., 2012; Maruyama et al., 2020; Newman et al., 2021). Therefore, the regulation of inflammation has become a key focus of bone healing. Previous studies have demonstrated the critical role of macrophages and bone marrow mesenchymal stem cells (BMSCs) in bone healing, coordinating inflammation and tissue regeneration (Loi et al., 2016; Wang Y H et al., 2022; Zhao et al., 2022). Macrophages are important intrinsic immune cells that have an active role in maintaining bone homeostasis (Kong et al., 2019; Schlundt et al., 2021). In addition, BMSCs can differentiate directly into osteoblast lineages, attract and recruit other cells, or create a microenvironment conducive to bone regeneration by secreting growth factors (Lin et al., 2019; Zhang et al., 2020). Furthermore, recent studies have shown that macrophages share a microenvironment with BMSCs, including cytokines, chemokines, transcription factors and signalling molecules that regulate bone healing. Osteoimmunology has become a hot topic of research in recent years (Walsh et al., 2006; Tsukasaki and Takayanagi, 2019). Therefore, this review will describe the role of macrophages and BMSCs in bone healing and provide insight into the crosstalk between the two types of cells, with the aim of providing new ideas to promote bone healing.

## 2 Macrophages in bone healing

### 2.1 Biology of macrophages

Macrophages are an inherent cell subset of the immune system, first proposed in the late 19th century by Elie Metchnikov, who described them as a group of immune cells devoted to phagocytosis (Metschnikoff, 1891). Macrophages play an important role in maintaining tissue homeostasis and promoting tissue repair by not only removing apoptotic cells and foreign or pathogenic substances through phagocytosis, but also by secreting a range of cytokines to form a pro- or anti-inflammatory microenvironment (Nobs and Kopf, 2021; Kloc and Kubiak, 2022). Macrophages are derived from monocytes, which are derived from precursor hematopoietic stem cells in the bone marrow. Monocytes mature in the bone marrow and then circulate in the blood (Arango Duque and Descoteaux, 2014). Circulating monocytes have several differentiation fates, including maturation into macrophages in response to injury or inflammation or migration into tissues to become resident macrophages (Mosser and Edwards, 2008). Among them, tissue resident macrophages are also derived from the embryonic yolk sac and maintain homeostasis in different tissues and organs, such as Kupffer cells in the liver, alveolar macrophages in the lung, and Langerhans cells in the skin (Bleriot et al., 2020; Nobs and Kopf, 2021). Bone and bone marrow contain specialized subsets of resident macrophages that contribute to skeletal biology and/or hematopoiesis. The resident macrophages present in the bone marrow are OsteoMacs, which account for 15%–20% of total bone marrow cells and exhibit F4/80, CD68, Mac3+and TRAP- (Sinder et al., 2015). They are mainly located near osteoblasts and support osteoclastogenesis and bone formation (Chang et al., 2008). OsteoMacs increase bone matrix deposition during fracture healing and interact with components of the hematopoietic microhabitat, including osteoblasts and megakaryocytes, to regulate the function of hematopoietic stem and progenitor cells (Alexander et al., 2011; Mohamad et al., 2017). Depletion of monocyte-derived macrophages and OsteoMacs leads to loss of osteoblasts in bone and mobilization of hematopoietic stem cells and progenitor cells (HSPC), thus making them key cellular components of the hematopoietic microhabitat (Winkler et al., 2010).

**FIGURE 1:**
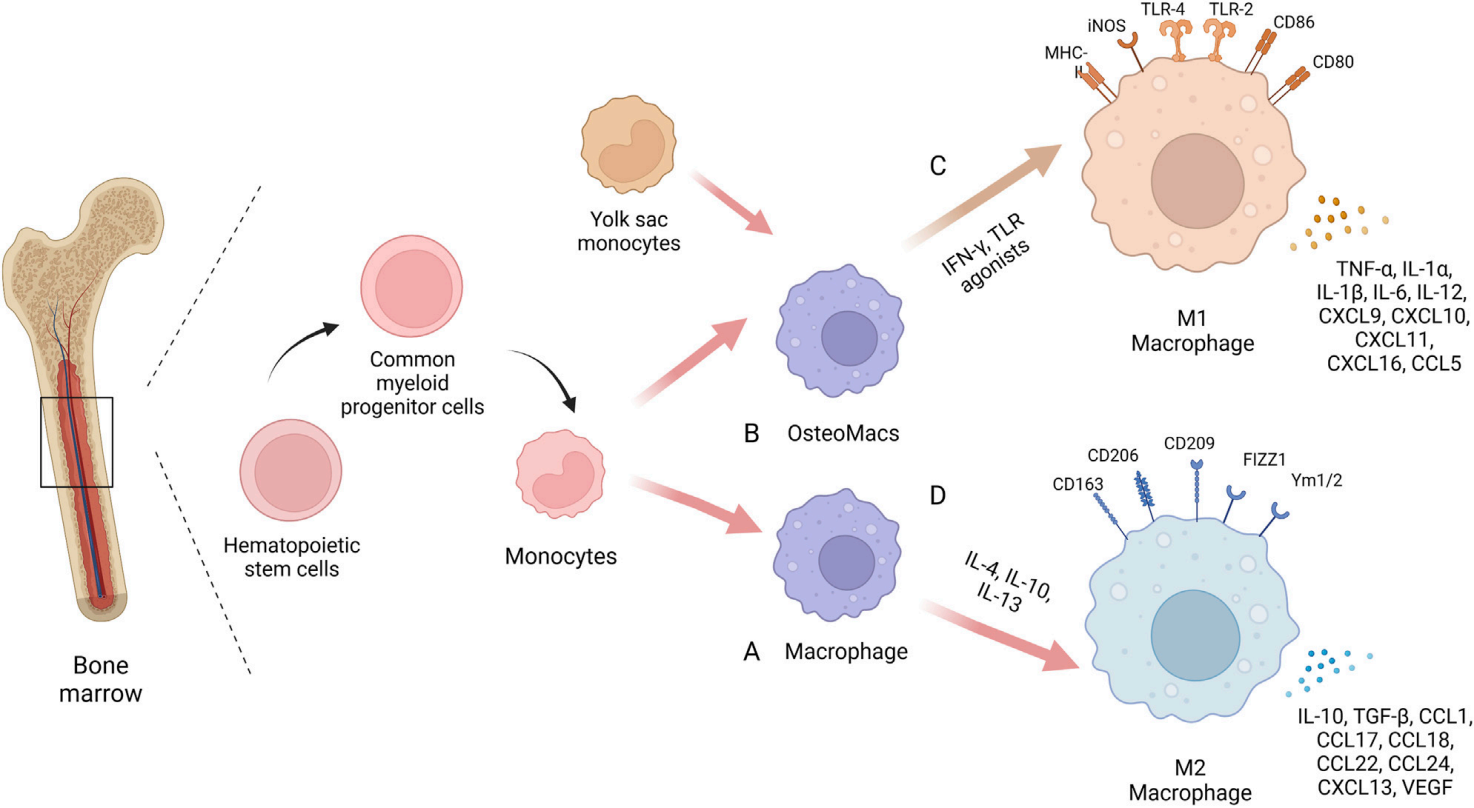
The biology of macrophages. In the same bone marrow ecological niche as stem cells, macrophages are derived from monocytes, which are derived from precursor hematopoietic stem cells in the bone marrow. Circulating monocytes can differentiate into mature macrophages **(A)** or migrate into bone tissue to become osteoMacs **(B)**. A proportion of these tissue resident macrophages also originate from the embryonic yolk sac. Macrophages have high plasticity and can be polarized into different phenotypes when faced with different microenvironmental stimuli and signaling cascades: “Classical activation” of M1 macrophages **(C)** and “alternate activation” of M2 macrophages **(D)**. Created with BioRender.com.

Macrophages have a high degree of plasticity. When macrophages face different microenvironmental stimuli and signal cascades, they can be polarized into different phenotypes: “classically activated” M1 macrophages that promote inflammation and “alternatively activated” M2 macrophages that promote tissue regeneration. Macrophages are more likely to polarize towards the M1 phenotype when stimulated by IFN-γ and TLR agonists such as LPS, while towards the M2 phenotype when stimulated by IL-4 or IL-13 (Murray, 2017). Recent studies have found that some non-cytokine extrinsic pathways such as hypoxia and lactate production can also promote macrophage polarization by regulating macrophage function and metabolism (Colegio et al., 2014; Liu et al., 2020; Noe et al., 2021). M1 macrophages express a variety of pro-inflammatory mediators, including TNF-α, IL-1β, and are accompanied by high expression of iNOS/CCR7/HLADR, which play a central role in host defense against infection, whereas the M2 macrophages express molecules such as Arg1, Ym1, IL-10 and CD206, among others, molecules that may be involved in tissue regeneration and tumour progression (Murray, 2017). In inflammatory diseases, M1 macrophages are enriched in early inflammatory sites, phagocytosing bacteria and apoptotic cell debris to protect the body from foreign substances. In the later stages of inflammation, M2 macrophage mainly play a role in suppressing inflammation, repairing tissue and rebuilding tissue structure. During the development of inflammation, the ratio of M1/M2 macrophage populations changes over time and the two phenotypes of macrophages can be interchanged, making them attractive targets for therapeutic intervention. Several studies have shown that targeting the macrophage phenotype to create a microenvironment conducive to tissue repair and regeneration can improve various diseases such as atherosclerosis, obesity, tumours, asthma and bone diseases (Liu et al., 2014; Eshghjoo et al., 2022; Goodman et al., 2022; Xiao et al., 2022) (Figure 1).

### 2.2 The relationship between macrophages and osteoclasts

The balance between osteoblasts and osteoclasts is important in the process of bone homeostasis. Osteoclasts are multinucleated giant cells that resorb bone and ensure the development and continuous remodeling of the skeletal and bone marrow hematopoietic ecological niche (Jacome-Galarza et al., 2019). It has been shown that defective osteoclast activity leads to osteosclerosis and bone marrow failure, while excessive activity can lead to bone loss and osteoporosis (Yoshida et al., 1990; Dai et al., 2002).

Osteoclasts are derived from myeloid progenitor monocytes or OsteoMacs with similar marker receptors on their surface, with osteoclasts being F4/80+/TRAP- while OsteoMacs and monocytes being F4/80+/TRAP-. Osteoclasts can be formed by differentiation of immature cells in the monocyte/macrophage lineage or by differentiation of mature bone macrophages (Udagawa et al., 1990). Among these, myeloid progenitor cells differentiate into bone macrophages upon stimulation by macrophage colony-stimulating factor (M-CSF) alone. Under the dual stimulation of M-CSF and nuclear factor κb receptor activator ligand (RANKL), myeloid progenitor cells differentiate into osteoclasts. rANKL and M-CSF are determinants of macrophage differentiation into osteoclasts (Yao et al., 2021). In addition, osteoclast differentiation is inhibited by osteoprotegerin (OPG), which is produced by osteoblasts and binds to RANKL, thus blocking the interaction with RANK (Lacey et al., 1998). Macrophage differentiation into osteoclasts requires several regulatory factors such as PPARg, ERRa, PGC-1b, NDUFS4 and maternal VLDLR (Yao et al., 2021).

The macrophage-osteoclast axis plays a crucial role in bone damage caused by inflammatory and immune diseases. In rheumatoid arthritis, monocytes differentiate into macrophages and osteoclasts and are involved in promoting synovial inflammation and joint damage. In addition, IL-6, TNF-a and IL-1β secreted by macrophages recruited to synovial tissue further increase osteoclast production (Gambari et al., 2020). In an ovariectomized osteoporotic mouse model, it was found that osteoclasts and macrophages were increased in both cortical and trabecular bone, while macrophages were shown to support osteoclast-mediated bone resorption by removing resorption by-products including bone particles and TRAP (Batoon et al., 2021). Therefore, targeting the macrophage-osteoclast axis is important for the treatment of bone injury.

### 2.3 The function of macrophages in bone healing

Bone healing is a complex biological and biomechanical process. The previous concept of the diamond four edges of bone healing believed that osteoprogenitor cells, growth factors, bone conduction scaffolds and mechanical environment jointly provide protection for bone regeneration and bone healing (Giannoudis et al., 2007). However, in recent years, more and more evidence has shown that the final fracture healing is highly dependent on the initial inflammatory phase, in which macrophages and their secreted factors play an important role in fracture healing (Singh et al., 2012; Tsukasaki and Takayanagi, 2019).

When injury occurs, the first event of healing is the formation of a fracture haematoma, where the blood vessels at the fracture site rupture and the haematoma microenvironment is altered. At the same time inflammatory factors are secreted in large numbers, initiating an inflammatory cascade response in which immune cells are recruited to the site of trauma and an acute inflammatory phase is thus initiated, peaking within the first 24–48 h (Goodman et al., 2019). Neutrophils, the first immune cells to arrive after injury, recruit a second wave of inflammatory cells to infiltrate the fracture site, namely, monocytes/macrophages, by secreting inflammatory factors and chemotactic mediators such as IL-6 and CCL2 (Boniakowski et al., 2018). In addition, resident macrophages within the bone tissue which are distributed among bone lining cells within bothendosteum and periosteum can also respond to injury stimuli and play a role in bone healing. Moreover, It has been reported that the depletion of macrophages in bone tissue affects bone repair. In the mice models of femoral fracture, the degree of callus formation correlates with the number of macrophages present within the callus (Batoon et al., 2019).

**FIGURE 2:**
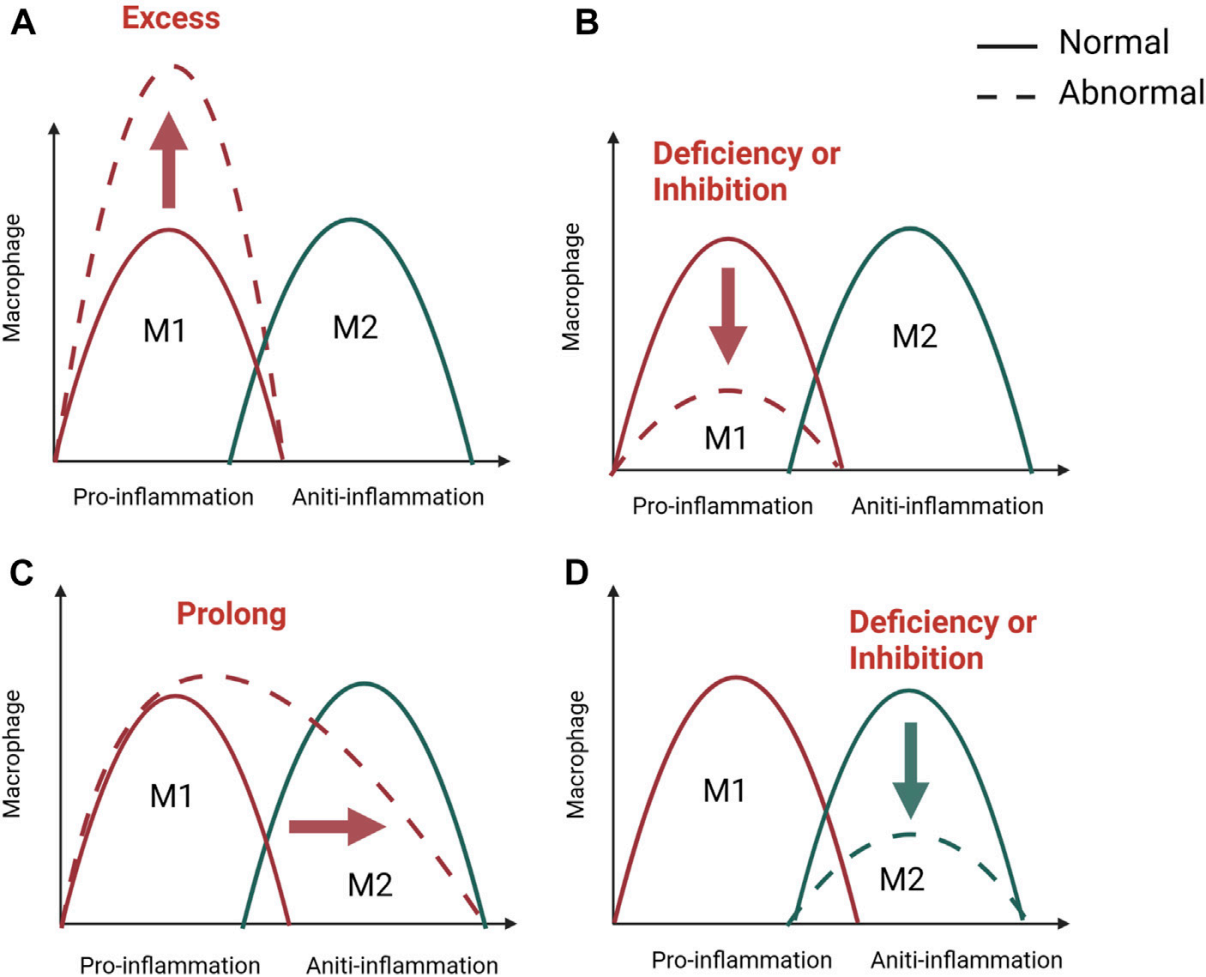
M1/M2 swich of macrophage in bone healing. The M1/M2 switch of macrophages is important for inflammation and bone regeneration. M1 macrophage dominate in the pro-inflammatory stage, while M2 macrophage in anti-inflammatory phase. Unsuccessful regeneration of bone fractures is associated with a excessive **(A)**, inhibited, **(B)** or prolonged **(C)** pro-inflammatory phase and a lack of anti-inflammation **(D)**. Created with BioRender.com.

In the early stages of inflammation, macrophages on the one hand phagocytose and remove microorganisms, necrotic tissue and temporary fibrin matrix. On the other hand, macrophages polarize to the M1 type in response to stimulation by the inflammatory environment and promote inflammation by releasing the cytokines TNF- α, IL- 6, and IFN-γ (Zhang et al., 2017; Pajarinen et al., 2019). Furthermore, the early and transient presence of M1 macrophages is known to recruit stem cells, promote osteogenic differentiation of stem cells and promote angiogenesis. *In vitro* experiments demonstrate that M1 macrophages can promote migration and osteogenic differentiation of human and murine stem cells by secreting cytokines such as TNF- α, OSM, BMP2 and BMP6 in inflammatory conditioned media (Nathan et al., 2019; Valles et al., 2020). At the same time, M1 macrophages can promote the initial formation of blood vessels by secreting VEGF (Qiu et al., 2020; Wang Y et al., 2022). Interestingly, a number of studies have shown that M1 macrophages are only involved in the early osteogenic phase and do not play a role in the later bone mineralisation phase (Vergadi et al., 2017; Pajarinen et al., 2019). In the later stages of inflammation and repair, macrophages can be converted from a pro-inflammatory M1 phenotype to an anti-inflammatory M2 phenotype, and M2 macrophages accelerate osteogenesis in the subsequent stages by secreting the osteogenesis-related proteins BMP-2 and TGF-β1. Besides, a series of anti-inflammatory cytokines such as IL-10 and IL-13 are also secreted to form a microenvironment conducive to tissue repair and bone healing (Zhang et al., 2017; Munoz et al., 2020; Liang et al., 2021; Schlundt et al., 2021). According to the above studies, M1 and M2 macrophages play different functions during bone regeneration. Macrophage phenotype switching affects the course of inflammation, and as in many other tissues, M1/M2 switching is important for bone regeneration (Figure 2). Failure of bone healing is associated with prolonged or amplified proinflammatory phase and lack of anti-inflammatory effects. It is believed that the presence of M1 macrophages for a long time or excessive aggregation can lead to prolonged chronic inflammation and impaired tissue regeneration (Maruyama et al., 2020; Newman et al., 2021). However, the early inflammatory response is necessary for bone healing and may also lead to failure of bone healing when inhibited during the acute inflammatory phase of bone healing. A systematic review of 47 animal researches by Al-Waeli found that non-steroidal antiinflammatory drugs reduced bone healing capacity (Al-Waeli et al., 2021). In addition, inflammatory bone diseases are often related to macrophage polarization, such as osteoporosis, osteoarthritis, periodontitis, diabetes-related bone diseases, and implant-associated inflammation (; Munoz et al., 2020; Bohaud et al., 2021). What’s more, impaired bone healing in aged rats has also been found to be associated with impaired M2 macrophage function (Gibon et al., 2016; Loffler et al., 2019). Therefore, appropriate adjustment of the M1 to M2 ratio by stimulating macrophages to enter a specific phenotype may be a necessary prerequisite for achieving better fracture healing efficiency.

### 2.4 Regulation of macrophages on osteogenic differentiation of BMSCs

Bone marrow mesenchymal stem cells are the main functional cells in the bone healing process and can differentiate into osteoblastic cell lineages. Also, after bone differentiation, the function and effectiveness of osteoblasts become critical in bone healing. There is growing evidence that macrophages can regulate the physiological functions of BMSCs and are important for the osteogenic differentiation of BMSCs, and the crosstalk between these two cell types deserves to be investigated in the field of bone regeneration. Bone marrow cultures from macrophage-deficient mice contain reduced numbers of mesenchymal progenitor cells, whose ability to differentiate into osteoblasts is further reduced (Vi et al., 2015). Moreover, macrophages have been shown to be required for osteoblast mineralization of the bone matrix (Chang et al., 2008).

Recent studies have confirmed that M1 and M2 phenotype macrophages regulate the biological behavior of BMSC. A series of *in vitro* studies revealed that enhanced osteogenic differentiation of BMSCs was observed after incubation of stem cells in conditioned medium (CM) derived from M1 macrophages. Lu et al. demonstrated that LPS-induced M1 macrophages promote BMSCs osteogenesis through the COX2-PGE2 pathway. Also, the presence of macrophages reduced OPG secretion, suggesting that macrophages may indirectly regulate osteoclast activity in addition to enhancing bone formation (Lu et al., 2017). Wasnik et al. found that inhibition of M1 macrophages with 1,25(OH)2D during the early proinflammatory phase resulted in impaired osteogenic potential of BMSCs, suggesting that M1 macrophages are important for the recruitment as well as osteogenic differentiation of BMSCs and such as TGF-β, VEGF and IGF-1 and are suspected to be the mechanism regulating osteogenic differentiation (Zhang et al., 2017). In addition, bone graft material can promote macrophage polarization towards M2 and promote BMSCs osteogenic differentiation, mainly due to high IL-10 expression (Shi et al., 2018). The high potential regulatory role of IL-10 in bone formation was further assessed in IL-10−/− knockout mice (Dresner-Pollak et al., 2004). These mice exhibited an osteoporotic bone phenotype characterized by reduced bone mass as well as reduced bone formation capacity compared to control wild-type mice. Moreover, in the vitro study of Gong et al. M2 macrophages enhanced osteogenic differentiation of MSCs, while M1 macrophages weakened it (Gong et al., 2016). However, in the study by Zhang et al. M0 and M1 macrophages stimulated osteogenic differentiation of MSCs exclusively through OSM and BMP2 in the early and middle stages. In contrast, in direct and indirect co-culture systems, M2 macrophages were more favorable for the mineralization of MSCs in the late stages of osteogenesis (Zhang et al., 2017). He et al. clearly illustrated how macrophage subtypes are involved in BMSCs osteogenesis. M0 macrophages significantly promoted osteogenic differentiation. M1 macrophages supported the proliferation of MSCs, while M2 macrophages promoted osteogenic differentiation of BMSCs. MSC incubated with CM from M2 macrophages exhibited an enhanced ability to form strong stem cell sheets (He et al., 2018). These studies demonstrate the important role of differentially polarized macrophages in bone regeneration.

## 3 Bone marrow mesenchymal stem cells in bone healing

### 3.1 Brief introduction of BMSCs and SSCs

BMSCs are stem cells with multidirectional differentiation potential and can differentiate into osteoblasts, chondrocytes, adipocytes and other cell lineages under specific induction conditions. Flow cytometry tests showed that the immunophenotypic marker subsets of BMSCs were CD73+/CD90+/CD105+/CD11b-/CD14-/CD34-/CD45-/CD19 -/CD79a-/human leukocyte antigen DR- (HLA-DR-) (Dominici et al., 2006; Boxall and Jones, 2012). Due to the ease of isolation and preservation of BMSCs, their homing properties, low immunogenicity, low tumorigenic risk, and low ethical controversy, they have shown broad applications in the field of cell therapy and regenerative medicine (Li et al., 2018).

Over time, cells with similar properties have been isolated from cord blood, adipose tissue, embryonic tissue, peripheral blood, and the liver (Zhou et al., 2019). However, recent studies have shown that *in vivo*, the transcriptome characteristics and differentiation potential of BMSCs from these different tissue sources differ significantly, and that allografts of these “stem cells” are often unable to form cartilage-bone structures or support a hematopoietic environment *in vivo*, revealing the complexity of BMSCs *in vivo* (Sacchetti et al., 2016; Zhang et al., 2023). To address these issues, the term “skeletal stem cells" (SSC) was introduced to denote the intrinsic, regenerative, and pluripotent cells of skeletal tissue that generate cartilage, bone, hematopoietic support matrix, and bone marrow adipocytes (Bianco et al., 2013; Li et al., 2022). Importantly, it has been advocated that in addition to colony-forming units (CFUs) and *in vitro* tri-lineage differentiation tests, self-renewal and pluripotency properties should be tested by rigorous *in vivo* assays, and serial transplantation studies are the gold standard for validating true SSCs (Bianco et al., 2008). However, further studies on the identification of SSCs are needed.

### 3.2 The function of BMSCs in bone healing

**FIGURE 3:**
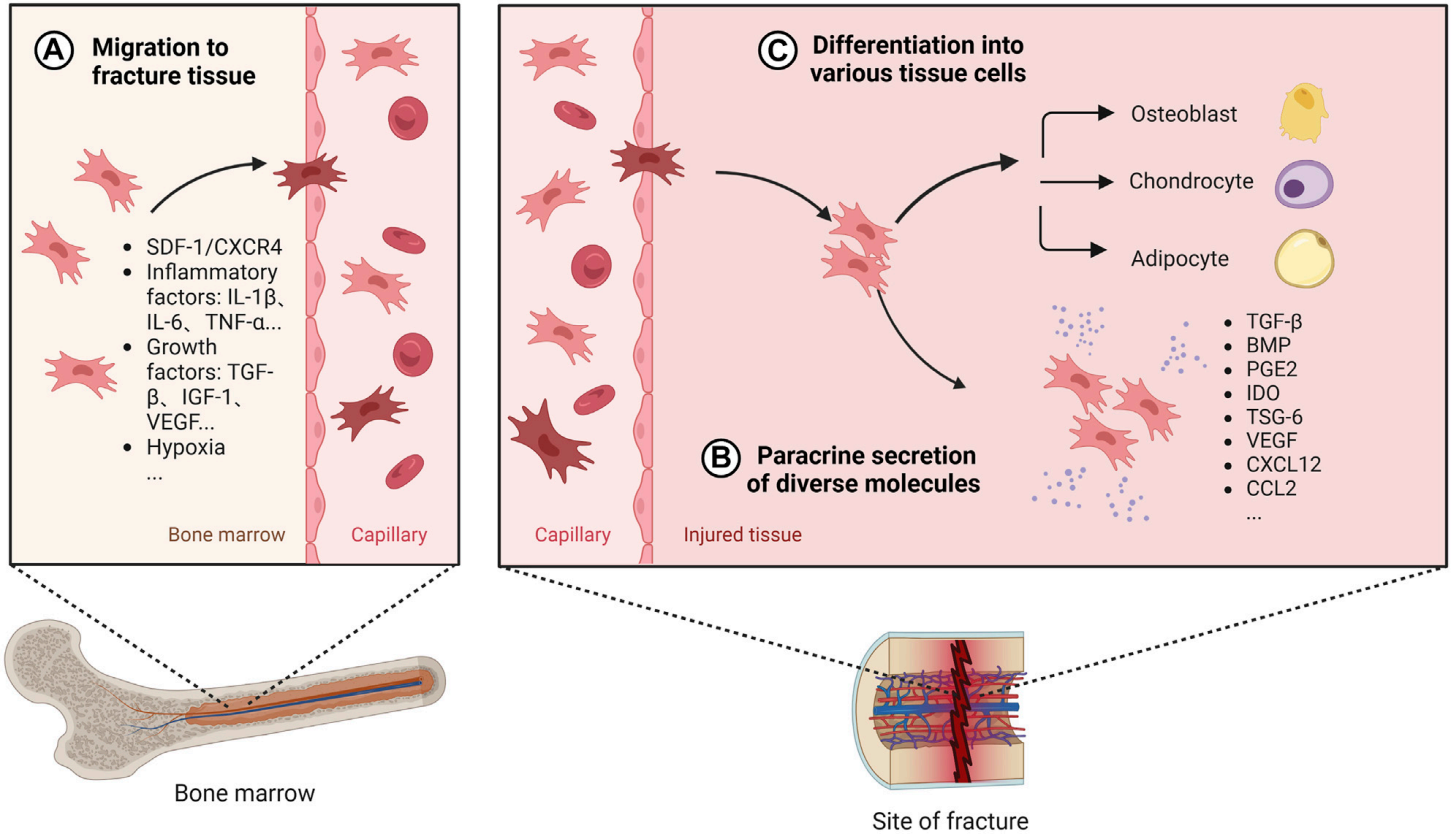
The role of BMSCs in bone healing. Once BMSC senses the signal released by the injured tissue, it will migrate from the bone marrow to the fracture site through the peripheral circulation, which is regulated by a variety of secretion factors and microenvironment **(A)**. After reaching the damaged tissue, BMSC releases diverse active factors to provide an appropriate environment for bone regeneration **(B)**. Also, BMSC can differentiate into different tissue cells to promote osteogenesis **(C)**. Created with BioRender.com.

BMSCs have been widely used in bone tissue repair and regeneration. Both cell therapy with direct addition of BMSCs and tissue engineering bone constructed with BMSCs as seed cells have been widely studied and applied, which are considered as the most promising methods for the treatment of bone defects (Zhang and Chen, 2017; Arthur and Gronthos, 2020). BMSCs promote osteogenesis mainly through recruitment at the site of injury, differentiation into osteoblastic cell lines and secretion of a range of mediators (Figure 3).

Under normal physiological conditions, BMSCs are mainly stored in cell niches in the bone marrow and in a resting state, with a low distribution in the peripheral blood and elsewhere. A dynamic balance is maintained between the mobilisation of BMSCs by bone marrow activation into the peripheral circulation and the homing of BMSCs to the bone marrow (Forte et al., 2006). The efficacy of BMSCs in cell therapy depends on their homing capacity and their ability to implant at the site of injury in the long term (Nitzsche et al., 2017). However, the transport of BMSCs from its ecological niche to the target tissue is a complex process and this delivery process is influenced by chemokines, cytokines, and growth factors. When a fracture occurs, the inflammatory response is triggered, the injury site is transiently hypoxic, and the expression of factors such as IL-1β, IL-6, TNF-α, OPN, TGF-β, IGF-1, and VEGF is elevated following bone tissue injury, which promotes the migration and viability of BMSCs (Xing et al., 2014; Lee et al., 2020; Mao et al., 2020; Feng et al., 2022). Furthermore related studies *in vivo* experiments in mice have shown that the SDF-1/CXCR4 signalling axis is one of the key chemokines mediating the recruitment of local and systemic-derived BMSCs. By controlling the release of SDF-1 not only does it promote better osteogenesis of exogenously delivered BMSCs in fracture and nonunion, but it also promotes endogenous cell recruitment of BMSCs at the site of injury (Chen et al., 2016; Zhang et al., 2021). BMSCs migration is also influenced by mechanical factors such as mechanical strain, shear stress, matrix stiffness, and microgravity (Fu et al., 2019). Therefore, targeting the ability to promote BMSC homing becomes one of the key aspects to promote bone healing.

After BMSCs reach the damaged tissue site, in addition to direct differentiation into osteoblasts, BMSCs can release many cytokines, growth factors, chemokines and exosomes through paracrine secretion under specific microenvironment, which can provide a good environment for promoting bone healing. Among them, TGF-β is a growth factor that can induce proliferation and differentiation of pluripotent stem cells, and BMP is a member of the TGF-β family, released by undifferentiated BMSCs to encourage cell differentiation into osteoblasts and promote osteoblast proliferation *in vivo* studies (Chen et al., 2012; Mohammed et al., 2021). BMP-2 binds to type I and type II serine-threonine kinase receptors, activates the Smad and mitogen-activated protein kinase (MAPK) pathways, and has significant osteogenic effects (Chen et al., 2012; Katagiri and Watabe, 2016). Currently, exogenously delivered BMPs are widely used in bone tissue engineering to further promote bone regeneration and bone healing (Kanczler et al., 2010; Decambron et al., 2017). However, it has also been suggested that BMPs facilitate the differentiation of MSCS into adipocytes *in vitro* (Futrega et al., 2018). Reestablishing the vascular network is a critical step in bone healing. VEGF, a potent growth factor involved in angiogenesis, has been extensively studied and shown to be involved in bone repair *in vivo*. During endochondral osteogenesis in a mouse fracture model, VEGF regulates angiogenesis, chondrocyte apoptosis, cartilage remodeling, and osteoblast migration (Deckers et al., 2002; Hu and Olsen, 2016a). It has been shown that BMPs stimulate VEGF expression in osteoblasts and osteoblast-like cells (Hung et al., 2007). BMSC upregulates the expression of VEGF and promotes the formation of vascular network, which is beneficial to increase oxygen supply to the injured tissue (Hu and Olsen, 2016b).

In the paracrine process of bone marrow MSCs, exosomes (Exos) are considered as essential mediators of intracellular signaling, which are extracellular vesicles with diameters ranging from 30 to 200 nm and are rich in various substances such as microRNA (miR) and proteins. In recent years, an increasing number of authors have reported the role played by exosomes during bone reconstruction (Zhou et al., 2022; Ju et al., 2023). *In vivo* experiments demonstrate that BMSCs promote osteoblast differentiation and proliferation by secreting specific exosomes that transport different bioactive proteins to the target cells (Qin et al., 2016). In addition they can be taken up by human umbilical vein endothelial cells (HUVECs) and contribute to the multiplication and migration of HUVECs *in vivo*, where miRNA-29a plays an important role (Lu et al., 2020). In clinical applications, BMSC-Exos can assist in the healing of inflammatory, metabolic, and hormonal bone diseases. In a rat osteoarthritis model miR-326 delivered by BMSC-Exos, which targets HDAC3 and STAT1//NF-κB p65, can inhibit cartilage atrophy, thereby ameliorating osteoarthritis (Xu and Xu, 2021). Moreover, miR-140-3p carried by BMSC-Exos alleviates bone degeneration and promotes bone regeneration by targeting Plxnb1 in diabetic rats (Wang N et al., 2022). In the bone marrow microenvironment of postmenopausal osteoporosis, miR-27a-3p and miR-196b-5p in BMSC-derived Exos mediate communication between osteoblasts and osteoclasts, and play an important role in coordinating bone formation and resorption (Lai et al., 2022). Exosomes are thought to have a wider range of applications in the field of bone repair as more study is done on them.

## 4 The crosstalk between macrophages and BMSCs

**FIGURE 4:**
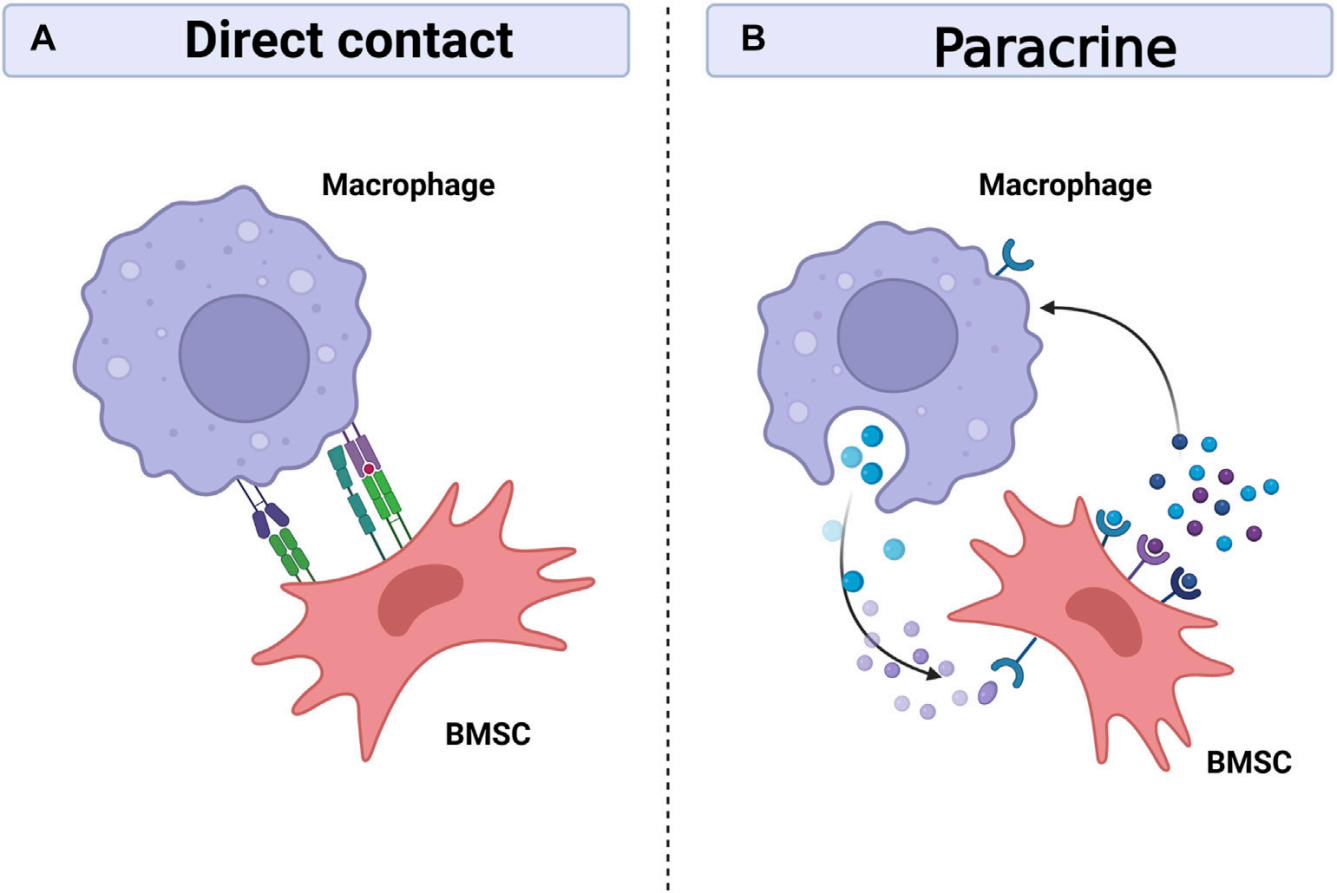
Interaction between macrophages and BMSCs. Macrophages can interact with BMSCs through direct contact between cells **(A)**, but the crosstalk mainly depend on paracrine between cells **(B)**. Macrophages and BMSCs promote bone healing by sharing many regulatory molecules, including soluble media, exosomes, signal molecules and transcription factors. Created with BioRender.com.

Bone regeneration requires the cooperation of multiple systems, and the skeletal and immune systems are gradually being recognized as critical in bone healing, as they share many regulatory molecules, including cytokines, receptors, signalling molecules and transcription factors. This has created an emerging discipline called osteoimmunology (Tsukasaki and Takayanagi, 2019). At the same time, the interactions between bone marrow mesenchymal stem cells and immune cells are becoming clearer. Prior research has demonstrated the capacity of BMSCs to control T cells, B cells, DC cells, and macrophages (English et al., 2008; Ghannam et al., 2010; Lee et al., 2014). On the one hand, BMSCs can regulate macrophage polarization, phagocytosis and metabolism (Adutler-Lieber et al., 2013; Vasandan et al., 2016; Jackson and Krasnodembskaya, 2017; Jin et al., 2019). On the other hand, macrophages, as regulated targets, also have feedback effects on BMSCs, including differentiation, migration, apoptosis and immunomodulatory functions (Li et al., 2019; Li et al., 2021; Lu et al., 2021; Maldonado-Lasuncion et al., 2021). Intercellular communication is necessary for bone healing, with cells communicating with each other either through direct contact or by secreting bioactive factors that diffuse through the intercellular space to neighbouring cells and are recognised and bound by the target cells thereby transmitting local information and influencing the functional activity of the cells (Turturici et al., 2014). It is currently thought that communication between macrophages and BMSCs is achieved mainly through direct cell contact and paracrine secretion (Figure 4).

### 4.1 Direct contact between cells

Macrophages can interact with BMSCs through direct cell-to-cell contact, although most reports suggest that the communication between them is mainly dependent on intercellular paracrine secretion (Mao et al., 2017; Mallis et al., 2020) (Table 1). The BMSCs extracted from human bone tissue *in vitro* studies were found to regulate immunity by activating STAT3 signalling through direct cell-to-cell interactions, thereby impeding the maturation of antigen-presenting cells (APCs), of which macrophages are one of the major antigen-presenting cells (Gur-Wahnon et al., 2009; Siniscalco et al., 2011). In addition, BMSCs and macrophages can interplay through direct contact in a mouse model of miscarriage,: on the one hand, BMSCs regulate the conversion of macrophages to M2 in a tumour necrosis factor-stimulated gene 6 (TSG-6)-dependent manner, thereby alleviating inflammation; on the other hand, macrophages upregulate CD200 expression on BMSCs to enhance their immunomodulatory ability (Li et al., 2019). Moreover, Takizawa et al. discovered that in the mouse model, ICAM-1/LFA-1 mediated direct connection between BMSCs and macrophages, and that following direct cell contact rather than indirect contact, macrophages were polarized towards the M2 type and expressed much more IL-10 (Takizawa et al., 2017). Interestingly, when implanted with biomaterials, the reciprocal immunomodulatory effect between BMSCs and macrophages is predominantly paracrine, and direct contact is not necessary for this effect (Sridharan et al., 2021).

**TABLE 1:** Cell-cell contact in the crosstalk between macrophages and BMSCs.

	Implicated biomolecules	Mechanism of action	Donor	Recipient	Result	References
Cell-cell contact	STAT3 pathway	STAT3 pathway activation	MSC	Antigen-presenting cells (e.g.,.macrophage)	Modulate DCs maturation; M2 macrophage swiching	Gur-Wahnon et al. (2009)
	TSG-6、CD200/CD200R	Increase the expression of TSG-6; Upregulate the expression of CD200 on the stem cells and CD200R on the macrophage	MSC	T cells、macrophage	Inhibit CD4 + T cell proliferation and promote macrophage switch to M2	Vasandan et al. (2016)
	ICAM-1/LFA-1	Increase the expression of ICAM-1/LFA-1	MSC	Macrophage	Promote macrophage among Lin + blood cells switch to M2	Siniscalco et al. (2011)

### 4.2 Paracrine secretion

It is now generally accepted that paracrine secretion is the main cause of mutual crosstalk between BMSCs and macrophages. Among these, soluble mediators and exosomes play a crucial role in BMSC-macrophage communication (Table 2).

**TABLE 2:** Paracrine secretion in the crosstalk between macrophages and BMSCs.

	Implicated biomolecules	Mechanism of action	Donor	Recipient	Result	References
Soluble factors	IL-1β	NF-κB pathway activation	Macrophage	MSC	Enhance migratory potential of MSC	Carrero et al. (2012)
	IL-6	STAT3 and MAPK signaling pathways activation	Macrophage	MSC	Enhance migratory potential of MSC	Rattigan et al. (2010)
	TNF-α	Increase the expression of VCAM-1 on MSCs	Macrophage	MSC	Enhance migratory potential of MSC	Xiao et al. (2012)
	CCL2, CCL3, CCL4, CCL5, CXCL2, CXCL10, CXCL16	Increase the expression of chemokines in macrophages	Macrophage	MSC	Accelerate MSC homing to facilitate bone formation	Wang et al. (2018), Lu et al. (2021)
	OSM、BMP-2	Increase the expression of OSM、BMP-2	Macrophage	MSC	Promote the proliferation and osteogenic differentiation of MSCs	Zhang et al. (2017)
	PGE2	COX-2-PGE2 pathway activation	MSC	Marophage	Enhance M2 macrophage polarization	Kuppa et al. (2022)
	IDO	Increase of the expression of IDO	MSC	Macrophage/monocytes	Induce the polarization of monocytes toward M2 macropage and reduce monocytes infiltration	Kuppa et al. (2022)
	TGF-β	Akt/FoxO1 pathway activation	MSC	Marophage	Promote macrophage polarization towards the M2-like phenotype; reduce pro-inflammatory cytokine levels; enhance the macrophage phagocytic ability	Liu J et al., 2019
	TSG-6	Increase of the expression of TSG-6	MSC	Macrophage	Promote macrophage polarization towards the M2-like phenotype	Kuppa et al. (2022)
	CCL2 and CXCL12	Upregulate IL-10 expression in CCR2+ macrophages	MSC	Macrophage	Induce Peritoneal Macrophage M2 Polarization	Giri et al. (2020), Galipeau (2021)
Exos	miR-222、miR-155	Inhibit anti-apoptotic gene Bcl-2; Negatively regulate the BMP signaling pathway	M1 Macrophage	MSC	Decrease BMSC viability and migration and increase BMSC apoptosis; Inhibit bone repair	Giri et al. (2020), Galipeau (2021)
	miR-378a、miR-5106	Upregulate BMP signaling; Target SIK2 and SIK3 genes	M2 Macrophage	MSC	Induce BMSC osteogenic differentiation	Giri et al. (2020), Galipeau (2021)

#### 4.2.1 Soluble mediators

Macrophages modulate the recruitment and osteogenic differentiation of BMSCs through the secretion of a range of cytokines. When injury occurs, a series of *in vivo* and *in vitro* studies found that macrophages release a series of pro-inflammatory factors such as IL-1β, IL-6 and TNF-α that can boost BMSCs recruitment (Rattigan et al., 2010; Carrero et al., 2012; Xiao et al., 2012). In addition, Wang et al. applied an *in vivo* mouse intramuscular implant model and found that a large number of chemokines CCL2, CCL3, CCL4, CCL5, CXCL2, CXCL10, CXCL16, SDF-1 released by macrophages have been associated with BMSCs migration and differentiation (Wang et al., 2018; Lu et al., 2021). In addition to immunomodulation, MSCs have osteogenic and adipogenic potential; this is why MSCs are an important cell population in bone healing. Diverse potential osteogenic factors have been shown to be involved in the interaction between different macrophage subtypes and MSCs. Zhang et al. found *in vivo* that M0 and M1 macrophages significantly facilitated hBMSCs osteogenic differentiation via the OSM signalling pathway during early and mid-inflammation; in contrast, M2 macrophages promoted osteogenesis by expressing BSP during late bone healing (Zhang et al., 2017).

Similarly, BMSCs can modulate their immune properties by secreting soluble factors acting on macrophages, such as PGE2, IDO, TGF-β and TSG-6. These cytokines play an important part in immunosuppression and the alleviation of inflammation and are key mediators in the regulation of macrophage polarization towards the M2 phenotype (Liu F et al., 2019; Kuppa et al., 2022). In addition, BMSCs secrete chemokines CXCL12 and CCL2, which alter macrophage phenotype to anti-inflammatory M2 macrophages while downregulating M1-specific markers (Giri et al., 2020; Galipeau, 2021).

#### 4.2.2 Exosomes

Exosomes are extracellular vesicles formed by invagination or endocytosis and contain various types of biological information (Zhang et al., 2020). Recent studies have found that exosomes play a crucial role in the communication between macrophages and BMSCs (Chuah et al., 2022).

Macrophage exosomes mediate bone regeneration in a rat calvaria defect model (Kang et al., 2020). However, it has also been reported that exosomes from M0, M1, and M2 macrophages may have different effects on BMSCs (Xia et al., 2020). This may be due to the fact that different information substances are contained in different exosomes. Our previous study *in vitro* found that macrophages in periodontitis can mediate inflammatory stimulation through exosome pathway and inhibit the osteogenic differentiation of BMSC, which is consistent with the results of Kang et al. in a rat cranial bone defect model. (Kang et al., 2020; Song et al., 2022). Qi et al. further found that M1 macrophages-derived Exos significantly reduced hBMSCs viability and migration and increased BMSCs apoptosis (Qi et al., 2021). In contrast, M2 macrophage-derived Exos are more dominant in promoting the osteogenic differentiation of BMSCs, and microRNA plays an important role in it. Li et al. found that M2-Exos from mice promoted osteogenic differentiation and inhibited adipogenic differentiation of BMSCs by up-regulating miR-690 *in vitro* (Li et al., 2021). Besides, Xiong et al. suggested that miR-5106 is highly enriched in M2-Exos and can be transferred to BMSCs to target SIK2 and SIK3 genes to promote osteoblast differentiation both *in vivo* and vitro in mice (Xiong et al., 2020). In addition, BMSCs have poor osteogenic differentiation ability under high glucose environment, and M2-Exo can activate Hedgehog signaling pathway to promote osteogenic differentiation of BMSCs under high glucose environment, suggesting that M2-Exo has therapeutic potential in diabetic fractures (Zhang et al., 2022).

## 5 Therapy targeting macrophage-BMSCs interaction to promote bone healing

Current treatments to promote bone healing mainly include surgical treatment, biotherapy, cell therapy, and tissue engineering technology. Stem cell-based tissue engineering technology mainly focuses on exploiting the bone regeneration potential of mesenchymal stem cells (Schlundt et al., 2018; Mick and Fischer, 2022). However, bone healing should be considered as a bone immune phenomenon, which is a balance between inflammation and regeneration. So far, targeting the communication between macrophages and BMSCs to promote bone healing has become a new hotspot in treatment, but there are few relevant reviews. We summarize the following approaches for both cells to regulate the inflammatory response during bone healing and promote bone healing by targeting the crosstalk between macrophages and BMSCs.

### 5.1 Regulate macrophages polarisation to facilitate BMSCs osteogenesis

Macrophages play a vital role in the inflammatory phase of bone healing and therefore precise regulation of macrophages and thus the inflammatory response is a major strategy to promote bone healing. Successful bone healing is largely dependent on the timely conversion of the M1 phenotype to the M2 phenotype, and prolonged presence of M1 macrophages results in prolonged immune responses, chronic tissue inflammation, poor BMSCs osteogenesis, delayed tissue healing and failure of biomaterial integration (Xie et al., 2020). Several studies have hypothesised the possibility of promoting bone regeneration by modulating macrophage polarisation. The potential of biomaterials to modulate immune cell function has been reported, and osteoimmunomodulatory biomaterials systematically modulate cell behaviour and the bone immune environment, thereby influencing bone regeneration (Claes et al., 2012; Newman et al., 2021). Currently, loading biologically active molecules or modifying physical and chemical properties are considered to be the main strategies to interfere with biomaterials and thus modulate the immune response. Wu et al. fabricated a PCL/PVP (polycaprolactone/polyvinylpyrrolidone) scaffold loaded with telmisartan for cell adhesion, tissue ingrowth and bone defect filling. This scaffold in the rat bone defect model modulates macrophage polarization towards the M2 type and displays osteogenic properties for BMSCs through activation of the BMP2-Smad signaling pathway (Wu et al., 2022). PEEK is a novel biomaterial and the modified PEEK scaffold material loaded with BMSC-Exos in the rat bone defect model can regulate macrophage M2 polarization and promote osteogenic differentiation of BMSCs through the NF-κB pathway (Fan et al., 2021). In addition, Tsuchiya et al. microroughened the surface of PEEK material and the modified PEEK scaffold resulted in higher proliferation and differentiation of BMSCs in rat vivo, while inhibiting the secretion of inflammatory factors by macrophages (Sunarso et al., 2018). These studies *in vivo* demonstrate that modified biomaterials exhibit excellent immunomodulatory properties, which in turn promote bone regeneration. However, most studies have mainly focused on the anti-inflammatory and regenerative role of M2 macrophages, while the important pro-inflammatory role of the M1 phenotype in the early inflammatory phase has been grossly underestimated. Baratchart et al. described the behaviour of macrophages during bone healing through integrated calculations, revealing the importance of macrophage polarisation time in bone healing (Baratchart et al., 2022). Based on this, scaffolds in tissue engineering have been modified accordingly, controlling the time points to sequentially promote the activation of the M1 and M2 phenotypes of macrophages and thus promote bone regeneration. In a recent research, a superparamagnetic hydrogel was developed to control macrophage polarisation in a timely manner, preserving the essential role of M1 macrophages in the early stages of tissue healing, while enhancing the role of M2 in tissue regeneration in the late stages of bone healing in a rat cranial bone defect model (Huang et al., 2022). In addition, Spiller et al. designed scaffolds that allow sequential delivery of cytokines, releasing M1 macrophage activators (e.g., IFN-γ) during the first 24 h, followed by factors that promote differentiation to M2 macrophages (e.g., IL-4). The precise timing of this macrophage polarization matches the regenerative process of the injured tissue, ultimately leading to the optimization of immunomodulated bone healing *in vivo* (Spiller et al., 2015).

### 5.2 Enhance the immunomodulatory ability of BMSC to macrophages

As the role of cellular communication between BMSCs and macrophages has been increasingly emphasized by researchers, the immunomodulatory effect of BMSCs on macrophages has also been suggested as an important target to promote bone healing (Kuppa et al., 2022). Modification of the secretion profile of BMSCs by pretreatment can enhance the immunomodulatory capacity of stem cells and promote tissue repair (Noronha et al., 2019).

MSCs can regulate the balance of pro- and anti-inflammatory factors in inflamed tissues, creating the necessary microenvironment for successful healing (Suzdaltseva et al., 2022). The immunomodulatory activity of BMSCs has been shown in numerous studies to be impacted by pro-inflammatory cytokines by pretreating the cells with substances that imitate the inflammatory environment to which BMSCs are exposed when they first penetrate sites of tissue injury and regeneration. This strategy aims to enhance immunosuppressive function and increase the secretion of its immunomodulatory factors (Kuppa et al., 2022). Currently, several studies *in vitro* have shown that IFN-γ pretreatment improves the immunomodulatory capacity of MSCs by promoting the expression of immunomodulatory molecules (e.g., PGE2, HGF, TGF-β, and CCL2) and proteins (e.g., annexin-A1, lactotransferrin, and aminopeptidase N) (Sunarso et al., 2018; Takeuchi et al., 2021; Yao et al., 2022). Pretreatment of mouse MSCs with TNF- α improves M2 macrophage polarization and prevents periodontal bone loss, which boosts the release of immunomodulatory substances such PGE2, IDO, and HGF (Noronha et al., 2019; Maruyama et al., 2020). However, it was found that pretreatment of MSCs with TNF-α or IFN-γ alone did not significantly reduce macrophage-mediated immune response and promote osteogenesis, whereas a combination of pro-inflammatory factors could achieve significant effects (English et al., 2007; Maruyama et al., 2020). Pretreatment of mouse BMSCs with IL-1β and IFN-ɣ *in vivo* significantly increased their potential to promote immune regulation: pretreated BMSCs inhibited M0 macrophage polarization toward M1 in the early stages of inflammation and promoted macrophage polarization toward the M2b phenotype by secreting IL-6 in the late stages of the anti-inflammatory response (Philipp et al., 2018). The combination of TNF-α and LPS could enhance the immunomodulatory properties of mouse BMSCs *in vitro* in response to macrophage polarization, while promoting the osteogenic differentiation ability of BMSCs (Lin et al., 2017). Furthermore, recent studies *in vitro* have shown that exosomes derived from hMSCs treated with a combination of TNF-α or IFN-γ synergistically promote anti-inflammatory M2 macrophage polarization by increasing the expression of CD73 and CCD5L, which becomes an effective cell-free therapeutic strategy (Watanabe et al., 2022). These researches demonstrate that BMSCs pretreated with pro-inflammatory factors can promote tissue regeneration by modulating macrophage polarization, and that the strategy of pretreating BMSCs can be applied to inflammatory bone disease and bone tissue engineering, as well as to chronic inflammatory diseases mediated by excessive macrophages. Therefore, we hypothesize that a series of pro-inflammatory factors secreted by macrophages early in fracture inflammation could further enhance the crosstalk between BMSCs and macrophages in bone healing. However, there are few *in vivo* studies on the efficacy of pretreatment of BMSCs with pro-inflammatory cytokines to make this possible, and further studies are needed in this area.

Another strategy that has been studied is to alter the BMSCs living environment through biological or physical stimuli, which may promote the release of more immunomodulatory factors from BMSCs (Li et al., 2015; Wu et al., 2017). To create an environment similar to the BMSCs niche, various 3D cell culture techniques have been widely studied in tissue engineering. In the three-dimensional environment, BMSCs can maintain good multi-directional differentiation potential, homing and migration ability, and immunomodulatory ability, and tend to produce more immunomodulatory factors such as TSG6, HGF and PGE2 (Bogers and Barrett, 2022). At present, BMSCs encapsulated in hydrogels is a popular method for bone tissue engineering. Hydrogels can mimic the natural extracellular matrix and allow the change of mechanical properties such as rigidity and hardness, thereby promoting the secretion of immune regulatory factors by BMSCs and enhancing bone tissue regeneration (Ji et al., 2020; Li et al., 2023). In the latest study, a 3D printed scaffold that could encapsulate both macrophages and BMSCs was applied to a rat skull defect model. The cells in the hydrogel were gradually released, and the communication between the two cells was enhanced, which effectively promoted the M2 polarization of macrophages and the osteogenic differentiation of BMSCs in the microenvironment of the bone defect (Noronha et al., 2019). In addition, under hypoxic growth conditions, BMSCs can secrete more immunomodulatory molecules such as IDO, IL-10 and PGE2 (Kadle et al., 2018) In the 3D spheroid cell culture structure, less oxygen may diffuse to the inner layer of the cells, and this hypoxic microenvironment promotes the release of immunomodulatory molecules from BMSCs. Moreover, Regmi et al. found *in vivo* that the survival ability of mouse BMSCs in the three-dimensional environment depends on autophagy and ROS mediated by HIF1A-HMOX1 axis related to hypoxia (Regmi et al., 2021; Fuentes et al., 2022; Sarsenova et al., 2022). The field of tissue engineering is developing rapidly. Creating a microenvironment suitable for BMSCs survival is very important for regulating the crosstalk between BMSCs and macrophages.

## 6 Conclusion

In this paper, we have reviewed the roles of macrophages and bone marrow mesenchymal stem cells in bone healing as well as the mechanisms and significance of their mutual crosstalk, revealing that bone healing is a complex process. This paper summarizes the mechanism of interaction between macrophages and BMSCs to promote bone healing based on the previous literature, which clarified that macrophages and BMSCs can interact with each other to promote bone healing. Macrophages and BMSCs coordinate inflammation and regeneration in bone healing. Macrophages can contribute to the differentiation and migration ability of BMSCs, while BMSCs can, in turn, immunomodulate macrophages. Therefore, how to regulate the crosstalk between BMSCs and macrophages provides a new therapeutic direction for optimizing bone healing.

In recent years, some biomaterials can modulate macrophage polarization toward M2 type to facilitate bone regeneration by loading bioactive molecules or improving physical and chemical properties, however, these studies neglected the precise timing of macrophage polarization in bone healing. Therefore, the design of biomaterials that can sequentially promote M1 and M2 phenotype activation at precisely controlled time points may be a future direction to optimize bone healing. In addition, the immunomodulatory effect of BMSCs on macrophages has been suggested as a major target for promoting bone healing. Pretreatment of BMSCs with pro-inflammatory factors or improvement of the BMSCs survival environment by biological scaffolds are both strategies to enhance the immunomodulatory ability of stem cells on macrophages. However, the volume ratio of BMSCs and macrophages in the co-culture system as well as the dose and stimulation time of pro-inflammatory factors need to be further investigated. We believe that these issues will be refined in the future to further effectively promote bone healing by targeting BMSC-macrophage interactions.
